# Bile Acids Increase Doxorubicin Sensitivity in ABCC1-expressing Tumour Cells

**DOI:** 10.1038/s41598-018-23496-y

**Published:** 2018-04-03

**Authors:** Simon Chewchuk, Tyler Boorman, Derek Edwardson, Amadeo M. Parissenti

**Affiliations:** 1 Ph.D. Program in Biomolecular Science, Laurentian University, Sudbury, ON P3E 2C6 Canada; 2 Health Sciences North Research Institute, Sudbury, ON P3E 5J1 Canada; 3 Division of Medical Sciences, Northern Ontario School of Medicine, Sudbury, ON Canada; 4 Division of Oncology, Faculty of Medicine, University of Ottawa, Ottawa, ON Canada

## Abstract

Tumour cells possess or acquire various mechanisms to circumvent the cytotoxic effects of chemotherapy drugs. One such mechanism involves the overexpression of ABC transporters that facilitate the extrusion of a variety of structurally distinct chemotherapy drugs from the cytoplasm into the extracellular space. While specific ABC transporter inhibitors have been developed, many affect other ABC transporters, particularly at elevated concentrations. It is also unclear whether they show clear efficacy for combatting drug resistance in cancer patients with minimal host toxicity. In this study, we demonstrate the ability of two bile acids [β-cholanic acid (urso-cholanic acid) and deoxycholic acid] to specifically inhibit ABCC1-mediated drug transport, augmenting doxorubicin accumulation in breast and lung tumour cells selected for doxorubicin resistance through overexpression of the ABCC1 (but not ABCB1) drug transporter. The bile acids could also restore uptake and sensitivity to doxorubicin in human endothelial kidney cells genetically engineered to overexpress the ABCC1 drug transporter. These observations suggest a previously unreported role for bile acids as ABCC1 inhibitors or regulators. Given its additional properties of minimal clinical toxicity in humans and its ability to inhibit aldo-keto reductases involved in anthracycline resistance and anthracycline-induced cardiotoxicity, β-cholanic acid merits further *in vivo* and clinical investigation.

## Introduction

Cytotoxic chemotherapy agents are still widely used to treat human cancers in both the neoadjuvant and adjuvant settings^1,2^. While combinations of cytotoxic and targeted chemotherapy drugs can be effective in improving patient survival, a major impediment to this approach is the presence of innate or acquired drug resistance mechanisms that circumvent the action of chemotherapy agents^3^.

Among the best described mechanisms of drug resistance are those associated with the elevated expression of one or more ATP-binding cassette (ABC) drug transporters^4^. These transporters, namely ABCB1, ABCC1 and ABCG2^5–7^, play a role in normal cell function, as they regulate cellular levels of a variety of small endogenous molecules that include cholesterol, its derivatives, and a variety of additional chemical substrates^5,6,8,9^. The ABC transporters, especially ABCB1, also function at the blood brain barrier to protect the brain from exposure to toxic agents^10^. Unfortunately, these transporters also circumvent the action of chemotherapy drugs by promoting the ATP-dependent efflux of drugs from the cytoplasm into the extracellular space^5^. In contrast to their clear role in drug resistance *in vitro*, it remains unclear whether they play a critical or central role in chemotherapy resistance for patients with solid tumours^11,12^. In contrast, their involvement in drug resistance, disease progression and clinical outcome for haematological malignancies is becoming increasing clear, particularly for acute lymphoblastic leukemia (ALL) and acute myelocytic leukemia (AML)^13,14^. The ABCB1 inhibitor tariquidar has been shown to increase sensitivity to doxorubicin, paclitaxel, etoposide, and vincristine in phase III trials of lung cancer. However, none of these trials were completed due to high systemic toxicity^15^. This illustrates the limited clinical utility of ABC transporter inhibitors to date.

While many ABC transporters are thought to function in a similar fashion, their substrate specificities and tissue distribution vary greatly^5,6,16,17^. ABCB1, also known as P-glycoprotein, is known to transport a large variety of commonly used chemotherapy agents, including the taxanes and anthracyclines^5^. ABCC1, also known as MRP1, transports a variety of chemotherapy agents, but transports taxanes very poorly^16^. While much has been accomplished in the development of specific and potent inhibitors of ABC transporters, few of these compounds have been applied to clinical trials, and those that have often result in off target toxicities, likely due to inhibition of ABC transporters not associated with the patients’ tumour. This highlights a weakness of targeting a single resistance factor alone.

Bile acids are a class of chemical compounds derived from the metabolism of cholesterol^18,19^. Their primary function is the solubilisation of fats and lipids during the digestive process^19,20^. In general, bile acids are produced in the liver and transported to the gallbladder where they are stored until needed^18,19^. The bile acids are then released into the small intestine where they form micelles with lipids. These micelles facilitate the absorption of lipids through the wall of the small intestine into the blood stream. Serum levels of bile acids vary greatly, from 0.3 µM to up to 3 mM, depending upon the tissue or organ in which they reside^20,21^. In addition to solubilizing lipids, bile acids also function as regulators of cholesterol metabolism by inhibiting enzymes of the aldo-keto reductase family. They also activate the farnasoid-X receptor, a transcription factor that regulates a variety of cellular functions^20,22–25^. While there is some evidence that bile acids can affect the expression of various ABC transporters through the farnesoid-X receptor^26,27^, in the present study we assessed the ability of bile acids to directly influence ABCC1 transport activity and ABCC1-mediated drug resistance. Previous studies have shown that membrane-perturbing agents and various bile acids such as deoxycholic acid and ursodeoxycholic acid can inhibit ABCC1-mediated transport of chemical agents in erythrocytes^28,29^. As we and other research groups have shown, some bile acids are known to interfere with other mechanisms of chemotherapy resistance (such as the ability of aldo-keto reductases to hydroxylate and detoxify doxorubicin). Thus, the prospect of targeting two or more resistance mechanisms simultaneously would be of interest in treating chemotherapy-resistant cancers^30–33^. Here we show the ability of β-cholanic acid, a known inhibitor of Akr1C3, to inhibit ABCC1-mediated drug transport. At concentrations normally used to inhibit Akr1C3, β-cholanic was able to inhibit ABCC1-mediated transport of doxorubicin. This same effect was not observed in cells where ABCB1-mediated drug transport is the primary mechanism of chemotherapy-resistance. We demonstrate that, even at high concentrations of deoxycholic acid and β-cholanic acid, doxorubicin retention is enhanced in chemotherapy-resistant cells that express the ABCC1 (but not ABCB1) drug transporter. This resulted in markedly improved doxorubicin sensitivity for cells expressing ABCC1, but not for cells expressing ABCB1.

## Results

### Augmented doxorubicin accumulation by bile acids in doxorubicin-resistant cells

**Figure 1:**
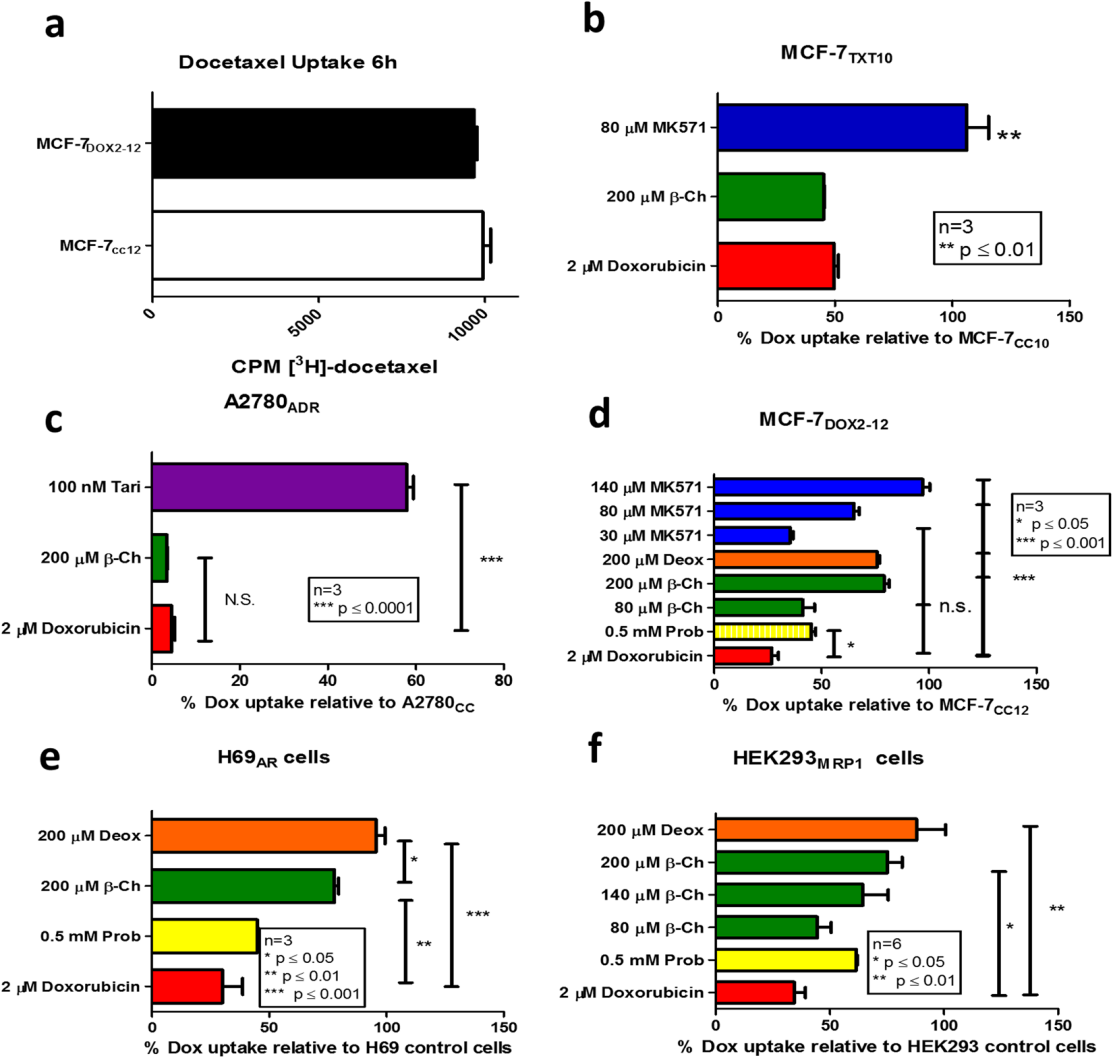
Measures of steady state uptake of doxorubicin or paclitaxel into various cell lines under various conditions. (**a**) Tritiated docetaxel uptake into MCF-7_CC12_ and MCF-7_DOX2-12_ cells. (**b**) Uptake of doxorubicin into MCF-7_TXT-10_ cells relative to MCF-7_CC10_ cells, as measured by flow cytometry. (**c**) Uptake of doxorubicin into A2780_ADR_ cells relative to A2780 cells, as measured by flow cytometry. (**d**) Effect of various agents on doxorubicin uptake into MCF-7_DOX2-12_ cells, relative to MCF-7_CC10_ cells. (**e**) Effect of various agents on doxorubicin uptake into H69_ADR_ cells, relative to H69 cells. (**f**) Effect of various agents on uptake of doxorubicin into HEK293 cells transfected with an ABCC1 expression vector relative to untransfected HEK293 cells. Agents included β-cholanic acid (β-ch, green), deoxycholic acid (deox, orange), MK571(blue), probenecid (prob, yellow) or Tariquidar (tari, purple). All experiments were performed in triplicate, with p-values being calculated using an ANOVA with Bonferoni post-hoc tests.

Since prior studies have shown that bile acids can inhibit ABCC1-mediated transport of chemical agents in erythrocytes^28,29^, we first assessed whether β-cholanic acid could also enhance drug accumulation into breast tumour cells. Cellular doxorubicin levels were monitored by flow cytometry, while taxane levels were quantified by liquid scintillation counting of [^3^H]-docetaxel in cells. These experiments were performed in a series of previously established cell line models known for their ABC transporter status^34,35^. As shown in Fig. 1a, ABCC1-overexpressing doxorubicin-resistant MCF-7_DOX2-12_ cells exhibited no change in [^3^H]-docetaxel accumulation relative to cells “selected” in the absence of doxorubicin (MCF-7_CC12_ cells). For this reason, no further tests were performed to measure docetaxel uptake in these cells. In contrast, when MCF-7_TXT10_ cells (known expressors of ABCB1, but not ABCC1) were assessed for their ability to uptake doxorubicin, these cells exhibited just 50% of the doxorubicin accumulation of MCF-7_CC12_ cells (Fig. 1). Interestingly, treatment of these cells with β-cholanic acid resulted in no statistically significant increase in doxorubicin accumulation (Fig. 1b). Similarly, the doxorubicin-resistant, ABCB1-expressing A2780_ADR_ ovarian cancer cell line exhibited only 4% of the doxorubicin uptake of parental A2780 cells and treatment of A2780_ADR_ cells with β-cholanic acid (at 200 μM) also had no effect on their ability to accumulate doxorubicin. Tariquidar (100 nM) (an ABCB1-specific inhibitor) restored doxorubicin accumulation in A2780_ADR_ cells to 58% of parental A2780 cells. This illustrates the role of ABCB1 as a primary mechanism of resistance to doxorubicin in MCF-7_TXT_ and A2780_ADR_ cells and the utility of tariquidar (but not β -cholanic acid) as an effective inhibitor of ABCB1-mediated doxorubicin resistance (Fig. 1c).

In contrast to the above observations, β-cholanic acid (at a 200 μM concentration) strongly restored sensitivity of ABCC1-expressing MCF-7_DOX2-12_ cells to doxorubicin and also increased doxorubicin accumulation (from 27% to 79% of drug-sensitive control MCF-7_CC-12_ cells). At 80 μM, β-cholanic acid restored doxorubicin accumulation to just 41% of control cells. A comparable restoration in doxorubicin accumulation was also observed when MCF-7_DOX2-12_ cells were treated with 200 μM deoxycholic acid (to 76% of control cells). The small molecule ABCC1 inhibitor MK571 (80 μM) was also effective at restoring doxorubicin accumulation to 65% of control cells, as expected. However, while MK571 is claimed to be a specific inhibitor of ABCC1, treatment of ABCB1-expressing MCF-7_TXT10_ cells with 80 μM MK571 resulted in significant restoration of doxorubicin uptake (Fig. 1b). This cell line does not have detectable ABCC1 expression^34^, suggesting lack of transporter specificity. The general organic anion transporter inhibitor probenecid (500 μM) only partially restored doxorubicin accumulation in MCF-7_DOX2-12_ cells (to 45% of doxorubicin-sensitive MCF-7_CC-12_ cells). At 140 μM, MK571 was more effective than 200 μM β-cholanic acid in restoring accumulation of doxorubicin (to 97% of control cells) (Fig. 1d). While both bile acids were capable of restoring uptake of doxorubicin, we chose to focus subsequent experiments on β-cholanic acid, as it is known to inhibit other resistance mechanisms detected in the MCF-7_DOX2-12_ cells, including the hydroxylation and inactivation of anthracyclines by the aldo-keto reductases^30–33^.

Bile acids were also shown to be effective at restoring accumulation of doxorubicin into doxorubicin-resistant ABCC1-expressing H69_AR_ lung cancer cells, with deoxycholic acid (200 μM) being slightly more effective than β-cholanic acid (200 μM) at restoring accumulation (95.5% and 87.8% of parental H69 cells, respectively; Fig. 1e). As a control, probenecid also restored doxorubicin accumulation to 45% of parental cells.

To determine if the observed effects of bile acids on doxorubicin uptake were the result of inhibiting ABCC1-mediated drug transport and not through effects on other molecules contributing to doxorubicin resistance in MCF-7_DOX2-12_ cells, HEK293 cells were stably transfected with an expression vector for ABCC1 and subsequently treated with doxorubicin and/or bile acids. In these HEK293_MRP1_ cells, doxorubicin accumulation was 34.4% of untransfected HEK293 cells (Fig. 1f). β-cholanic acid (at 80 μM and 140 μM) augmented doxorubicin accumulation in HEK293_MRP1_ cells to 44.4% and 64.4% of untransfected cells, respectively (Fig. 1f). At 200 μM, both β-cholanic acid and deoxycholic acid increased doxorubicin levels in HEK293_MRP1_ cells to 75.2% and 88% of untransfected cells, respectively. Probenecid (500 μM), a general organic anion transport inhibitor, caused a restoration of doxorubicin accumulation to 61.5% of untransfected cells.

**Figure 2:**
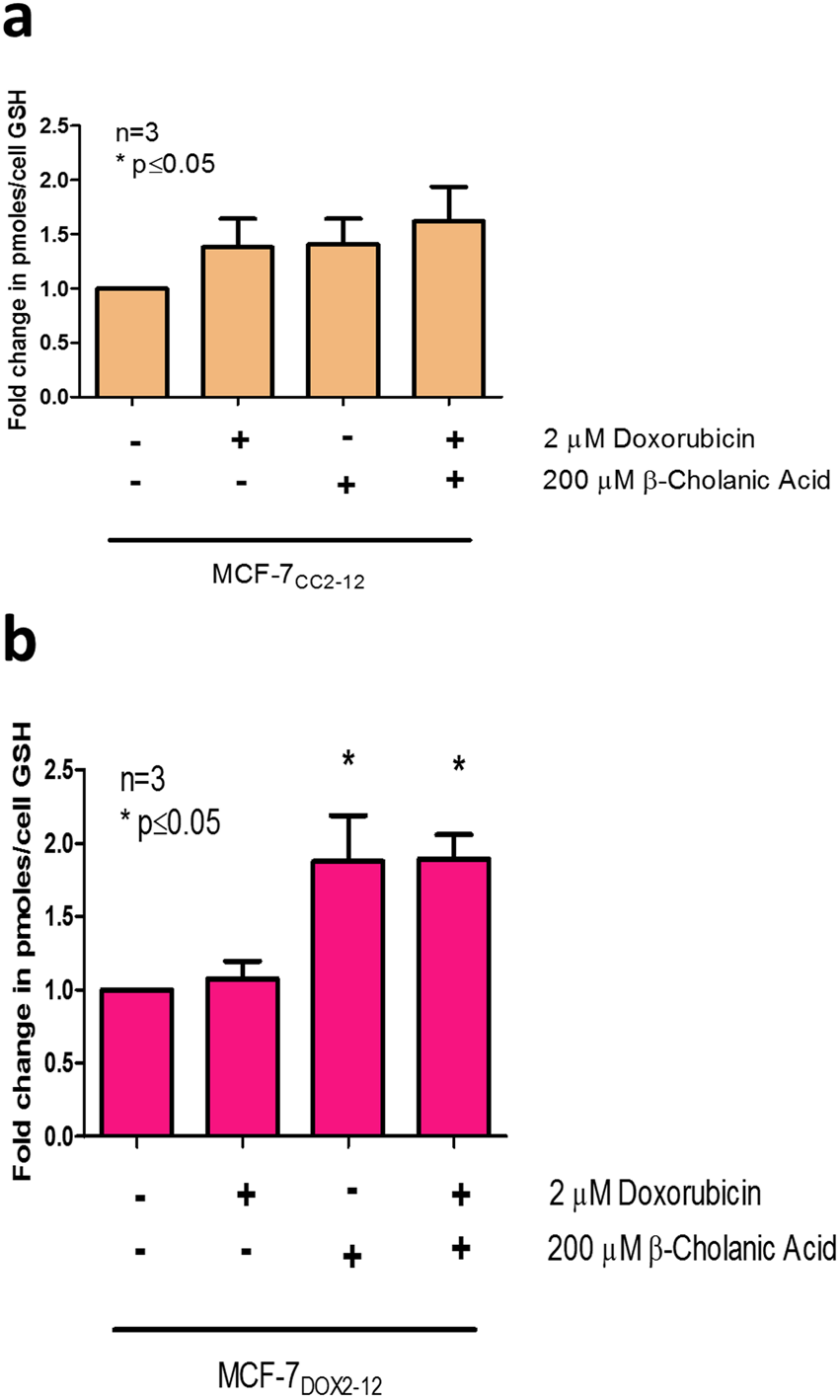
Effect of doxorubicin and β-cholanic acid on glutathione levels in buthionine-treated MCF-7 cells. MCF-7_CC12_ cells (**a**) and resistant MCF-7_DOX2-12_ cells (**b**) were treated simultaneously with buthionine sulfoximine (50 μM for 1 h) followed by combinations of doxorubicin (2 μM) and β-cholanic acid (200 uM) for assessment of their glutathione levels. Treatment with β-cholanic acid in MCF-7_DOX2-12_ cells resulted in elevated cellular glutathione levels. All data were standardized to respective untreated controls. Experiments were performed in triplicate. Statistical analyses were conducted using an ANOVA with a post-hoc Bonferoni correction.

ABCC1-mediated transport of doxorubicin has been shown to require the presence of glutathione^36^. Consequently, we also measured glutathione levels in MCF-7_DOX2-12_ cells in the absence or presence of β-cholanic acid. Cells were first pre-treated with buthionine sulfoximine, which irreversibly inhibits glutathione biosynthesis, preventing cells from replenishing glutathione during the course of the experiment. As shown in Fig. 2, MCF-7_CC12_ cells showed no significant change in glutathione levels under any treatment conditions. In contrast, MCF-7_DOX2-12_ cells showed significantly increased levels of intracellular glutathione when treated with β-cholanic acid (1.9-fold higher than untreated MCF-7_DOX2-12_ cells). This same increase (1.9-fold) was observed in MCF-7_DOX2-12_ cells treated with both β-cholanic acid and doxorubicin.

### Bile acids increase sensitivity to doxorubicin in doxorubicin-resistant cells

**Figure 3:**
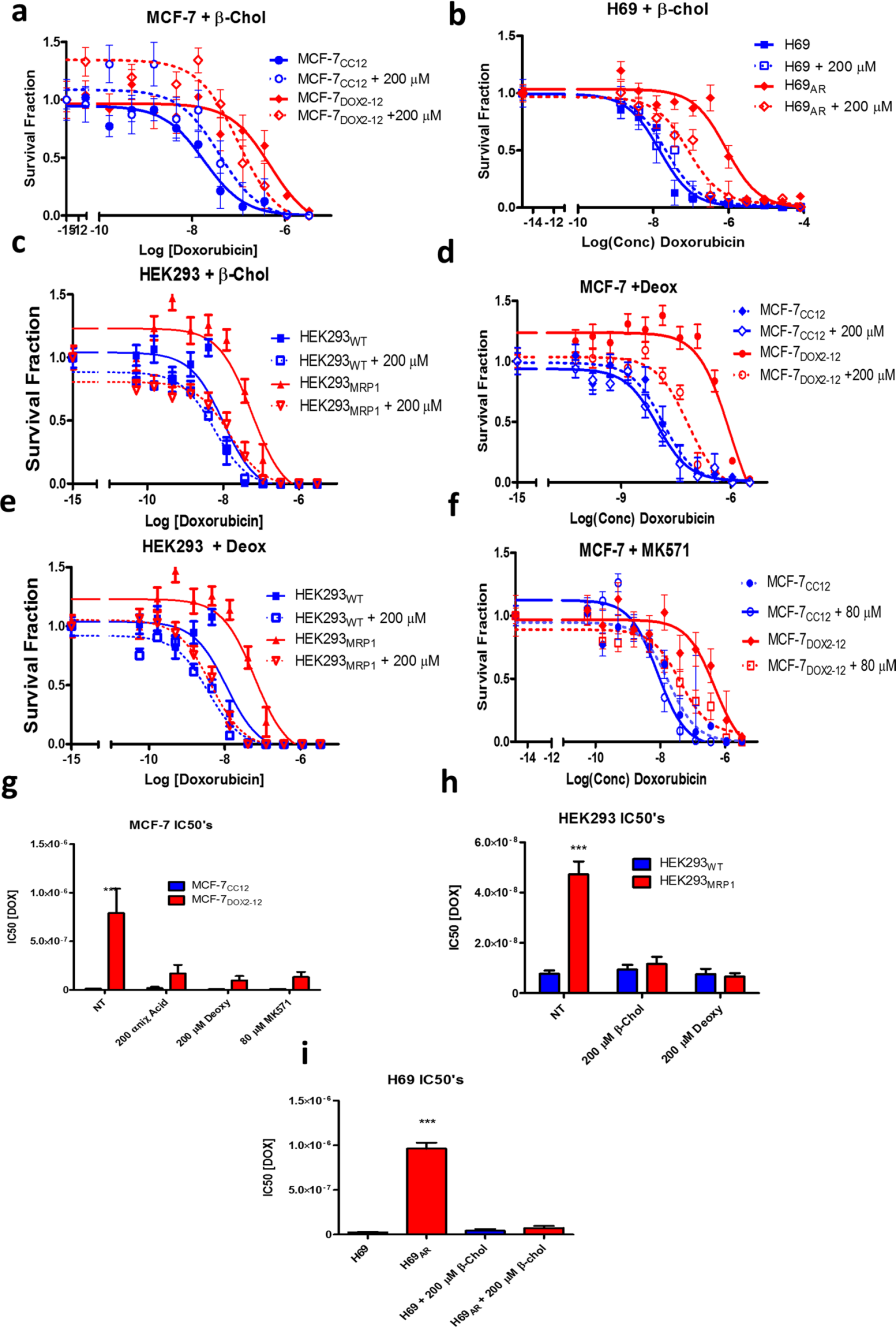
Effect of bile salts on sensitivity of human tumour cell lines to doxorubicin as measured using clonogenic assays. Cell lines used included MCF-7_CC12_, MCF-7_DOX2-12_, H69, H69_AR_, HEK293 and HEK293_MRP1_ cells. (**a**–**c**). Survival curves for MCF-7, H69 and HEK293 cells (and their doxorubicin-resistant counterparts) treated with increasing concentrations of doxorubicin in the presence or absence of 200 μM β-cholanic acid. (**d**,**e**). Doxorubicin sensitivity curves for MCF-7 and HEK293 cells as a function of doxorubicin concentration in the absence or presence of 200 μM deoxycholic acid. (**f**). Effect of MK571 on doxorubicin sensitivity in wildtype and doxorubicin-resistant MCF-7 cells. (**g**–**i**) Summary of IC50 values for doxorubicin for various cell lines under various conditions. *** p < 0.001 as determined by an ANOVA with a Bonferoni correction.

Since increased uptake of doxorubicin would be expected to increase the cytotoxicity of the drug, the effect of bile acids was also assessed on doxorubicin sensitivity in doxorubicin-sensitive and -resistant breast tumour cell lines (MCF-7_CC12_ and MCF-7_DOX2-12_ cells, respectively) and doxorubicin-sensitive and -resistant lung tumour cell lines (H69 and H69_AR_ cells, respectively). Each of the above drug-resistant cell lines express elevated levels of ABCC1. Using clonogenic assays, MCF-7_DOX2-12_ cells were found to exhibit increased doxorubicin sensitivity in the presence of the bile acids, with IC50 values for doxorubicin of 790 nM for untreated cells and 170 nM and 98 nM for cells exposed to 200 μM β-cholanic acid and 200 μM deoxycholic acid, respectively. This represented 4.6- and 8-fold reductions in the IC50 value, respectively. In contrast, the IC50 value for MCF-7_CC-12_ cells was 14.3 nM (Fig. 3f). These changes represent a shift in resistance factor from 55.2 in untreated resistant cells to 11.9 and 6.9 for β-cholanic and deoxycholic acid treated cells, respectively. Similarly, doxorubicin-resistant H69_AR_ cells exhibited restored sensitivity to doxorubicin, with IC50 values of 960 nM and 73 nM (13.2 fold reduction) for untreated and β-cholanic acid-treated cells, respectively. The IC50 value for wildtype H69_AR_ cells was of 24.1 nM (Fig. 3e,h). This corresponds to a resistance factor for doxorubcin of 3 in the presence of β-cholanic acid-treated cells compared to a resistance factor of 39.8 for untreated resistant cells (when compared to control H69 cells).

**Figure 4:**
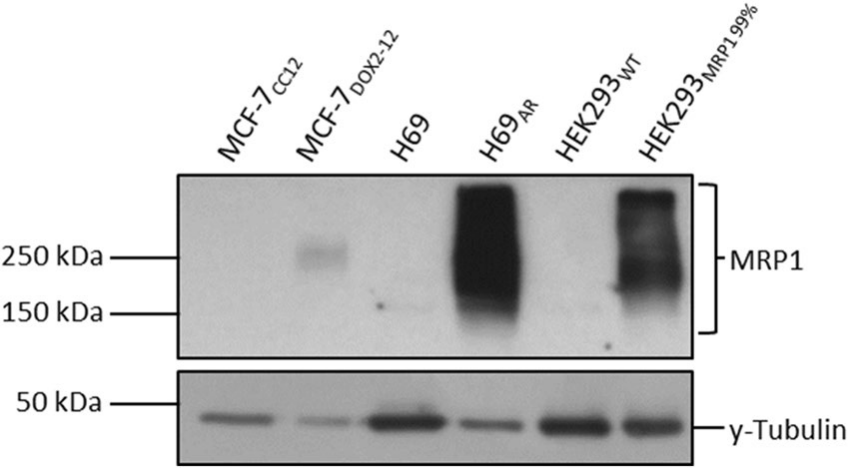
Representative western blots of ABCC1 levels in various cell lines, including drug-sensitive MCF-7_CC12_ breast and H69 small cell lung tumour cell lines and their doxorubicin-resistant counterparts (MCF-7_DOX2-12_ and H69_AR_ cell lines, respectively). ABCC1 levels were also assessed in HEK293 cells and HEK293 cells transfected with an ABCC1 expression vector.

To determine if the effects of β-cholanic acid on doxorubicin sensitivity were limited to cells selected for anthracycline resistance, ABCB1-expressing MCF-7_TXT10_ cells were also treated with doxorubicin and/or β-cholanic acid. Unlike the above doxorubicin-resistant cells lines, MCF-7_TXT10_ cells exhibited no change in doxorubicin sensitivity in the presence of the bile acid (data not shown). This suggested that β-cholanic acid does not potentiate doxorubicin cytotoxicity in all drug-resistant cell lines and may demonstrate selectivity towards cells overexpressing ABCC1. Hembruff *et al*. showed that MCF-7_TXT_ and MCF-7_EPI_ resistant cells express high levels of ABCB1, while MCF-7_DOX2-12_ cells express high levels of ABCC1 transporter^34^. These cell lines were developed in our laboratory by selection for survival in increasing drug concentrations and all are isogenic with parental MCF-7 cells. Cells were also “selected” in the absence of drug to control for increasing passage number (MCF-7_CC_ cells). MCF-7_DOX2-12_ cells, however, do not express the very high levels of ABCC1 seen in H69_AR_ cells (Fig. 4). Thus, the differing levels and types of drug transporters may account for the contrasting ability of β -cholanic acid to increase cellular sensitivity to doxorubicin in the above drug-resistant cell lines. Moreover, β-cholanic acid may selectively affect cells overexpressing ABCC1, given that ABCB1-overexpressing doxorubicin-resistant A2780_ADR_ cells did not exhibit increased doxorubicin sensitivity in the presence of β-cholanic acid, while ABCC1-overexpressing doxorubicin-resistant ABCC1-overexpressing cells did.

To test this hypothesis, HEK293 cells and ABCC1-transfected HEK293_MRP1_ cells were examined for their sensitivity to doxorubicin in the absence or presence of bile acids. As seen in Fig. 3c and e, both β-cholanic acid and deoxycholic acid augmented doxorubicin cytotoxicity in HEK293_MRP1_ cells. HEK293_MRP1_ cells exhibited an IC50 for doxorubicin of 47 nM in the absence of bile acids and IC50 values of 12 nM and 6.6 nM in the presence of β-cholanic acid and deoxycholic acid, respectively. This represented reductions in IC50 values for doxorubicin of 3.9-fold and 7.1-fold, respectively. Thus, IC50 values in the presence of bile acids were the range observed for wild type HEK293 cells (7.7 nM). Resistance factors were thus reduced to 1.5 and 0.85 for β-cholanic acid- and deoxycholic acid-treated cells, respectively.

As part of our investigation, we examined the effect of β-cholanic acid (200 μM) on cell viability (as measured by cell number). In one instance, β -cholanic acid did induce a reduction in colony counts in the clonogenic assay. Colony counts for MCF-7_DOX2-12_ cells were significantly reduced by 200 μM β-cholanic acid from 23.1 ± 0.9 colonies per field to 13.7 ± 0.5 colonies per field (Student’s paired T test; p = 0.0001). However, for other cell lines tested, the reduction in colony counts was not found to be statistically significant. MCF-7_CC12_ cells had colony counts of 39.7 ± 9.8 and 28.2 ± 1.1 in the absence and presence of β-cholanic acid, respectively (p = 0.29). H69 and H69_AR_ had colony counts of 167 ± 32.7 and 102 ± 28.7 in the absence of β-cholanic acid, respectively, while in the presence of the bile acid, colony counts were 103 ± 34 and 34 ± 8.6. P values for the effect of β-cholanic acid on colony number were 0.25 and 0.09 for H69 and H69_AR_ cells, respectively. Taken together, these observations with tumour cell lines suggest a trend that β-cholanic acid reduces tumour cell viability, and this effect may be greater for drug-resistant cells. However, β-cholanic acid had little to no effect on colony counts for non-transformed HEK293 or HEK293 cells transfected with the *ABCC1/MRP1* expression vector, despite the ability of the bile acid to augment doxorubicin cytotoxicity in the latter cell line. HEK293 cells had mean colony numbers of 112 ± 3.5 and 104 ± 6.7 in the absence and presence of β-cholanic acid, respectively (p = 0.36), while HEK293_MRP1_ cells exhibited 41.6 ± 4.0 and 41.1 ± 3.2 colonies, respectively (p = 0.94). Taken together, these findings suggest that the potentiation of doxorubicin cytotoxicity by β-cholanic acid is unrelated to its own moderate cytotoxicity towards tumour cells, as some cells exhibit little to no reduction in cell number in the presence of high concentrations of the bile acid. It is also noteworthy that despite the effect of β-cholanic acid on cell number in some cell lines, the bile acid has little consistent effect on doxorubicin sensitivity in any of the drug-sensitive cell lines tested (Fig. 3). In addition, β-cholanic acid had no ability to augment doxorubicin uptake into doxorubicin-resistant or docetaxel-resistant cell lines that lack ABCC1 expression (Fig. 1). This suggests that the expression of ABCC1 is critical to the ability of β-cholanic acid to potentiate doxorubicin cytotoxicity.

## Discussion

In this study, we provide strong evidence that bile acids can selectively reduce doxorubicin accumulation into ABCC1-expressing (but not ABCB1-expressing) tumour cells. This results in a strong promotion of doxorubicin sensitivity in doxorubicin-resistant tumour cells, providing they express the ABCC1 drug transporter. However, of the two bile acids, β-cholanic acid is of particular interest, due to its previously reported ability to inhibit aldo-keto reductases^31^. Several studies have shown that that the aldo-keto reductases play significant roles in anthracycline resistance in tumour cells. AKR1C3 can induce the hydroxylation and inactivation of doxorubicin^37^. Moreover, our laboratory and others have shown that aldo-keto reductases are elevated as cells acquire resistance to doxorubicin^30,38^. Interestingly, the 13-hydroxylated form of doxorubicin (doxorubicinol) exhibits strongly reduced cytotoxicity and DNA binding, as well as altered subcellular localization^32^. Zhong *et al*. showed that overexpression of recombinant aldo-keto reductase 1C3 in breast tumour cells was sufficient to induce doxorubicin resistance^38^. The efficacy of another anthracycline (daunorubicin) was also shown to be substantially reduced by the induction of aldo-keto reductases 1C1 and 1C3 in U937 leukemic cells^39^. However, potentiation of doxorubicin cytotoxicity by β-cholanic acid in our study did not appear to be through inhibition of drug hydroxylation because another bile acid (deoxylcholic acid), which has no aldo-keto reductase inhibitory activity^40^, was able to potentiate doxorubicin cytotoxicity at concentrations identical to β-cholanic acid.

Inhibition of aldo-keto reductase activity by β-cholanic acid may also have another attractive feature. Since it is the doxorubicinol metabolite that appears responsible for doxorubicin’s cardiotoxicity^41,42^, β-cholanic acid may reduce cardiotoxicity in patients administered doxorubicin. Interestingly, the P-glycoprotein inhibitor nilotinib was shown to significantly enhance doxorubicin’s ability to inhibit tumour xenograft growth in mice, but also increased doxorubicinol accumulation in the heart and cardiotoxicity^43^. Thus, effective strategies are necessary to block systemic toxicity, if drug accumulation is improved by ABC transporter inhibitors. β-cholanic acid may help in this regard.

In a recent study, we showed that while β-cholanic acid could inhibit the conversion of estrone to estradiol through inhibition of aldo-keto reductase activity, neither of two endogenous aldo-keto reductases (Akr1c3 and Akr1b10) appeared to play a significant role in doxorubicin resistance in the MCF-7_DOX2-12_ cell line^33^. Thus, our observations suggest another potential effect of β-cholanic acid in breast cancer. The drug could block estrogen-dependent growth and survival pathways in estrogen receptor-positive tumours.

Another attractive feature of bile acids are there minimal toxicities *in vivo*. Despite a trend towards reducing colony counts *in vitro* in the clonogenic assay (see Results), bile acids (including β-cholanic acid) are well tolerated in patients, even at a dose of 100 mg/day in infants^44^ and 10 mg/kg/day in adults^45^. The only toxicity seen at even higher doses (15 mg/kg/day) is diarrhea. According to https://clinicaltrials.gov, bile acids are currently being employed in a number of human clinical trials. Thus, these compounds merit further investigation, despite their lower affinity for ABCC1 compared to other small molecule inhibitors. The blood brain barrier is rich in ABCB1 expression^8^, but unlike MK571, β-cholanic acid would not be expected to inhibit this drug transporter. By inhibiting ABCC1 specifically, β-cholanic acid might augment accumulation of doxorubicin in ABCC1-expressing tumours, while retaining ABCB1’s ability to protect sensitive brain tissues to the damaging effects of doxorubicin.

It should be noted that the potency of bile acids in our hands was approximately 2.5-fold lower than that of a well-known ABCC1 inhibitor, namely MK571. The concentrations of bile acids used, however, were well within the physiological range of bile acids in humans^19^. Moreover, while MK571 was more potent than bile acids at increasing doxorubicin uptake into ABCC1-expressing doxorubicin-resistant tumour cells in our study, our findings also show that at a lower but equally effective concentration of MK571 (80 μM) also increased doxorubicin uptake into docetaxel-resistant MCF-7_TXT10_ cells (Fig. 1). These cells express both the ABCB1 and ABCC2 drug transporters^34^. Molinas *et al*. also showed that MK571 (as well as cyclosporine A and probenecid) inhibited ABCB10 transport activity^17^. We found that probenecid, another inhibitor of ABCC1, was also able to partially restore doxorubicin uptake into doxorubicin-resistant cells expressing ABCC1, but at a concentration more than double that of β-cholanic acid. This suggested that β-cholanic acid is a more potent ABCC1 inhibitor than probenecid in the presented model.

While bile acids appear to be specific for inhibition of ABCC1 over ABCB1, it should be noted that we did not examine the possible effects of bile acids on other ABC transporter proteins. Since doxorubicin uptake in MCF-7_TXT10_ cells was unaffected by β-cholanic acid (Fig. 1b) and these cells express both ABCB1 and ABCC2^34^, it is also likely that β-cholanic acid does not inhibit ABCC2 transporter activity. The restoration of doxorubicin uptake and cytotoxicity in cells overexpressing exogenous ABCC1 (HEK293_MRP-1_ cells) further suggests that the effect of bile acids in sensitizing cells to chemotherapy is primarily due to their ability to affect ABCC1 transport and not through potential additional effects on other proteins implicated in doxorubicin resistance.

The selectivity of bile acids for inhibition of ABCC1 transport activity (with little effect on ABCB1-mediated drug efflux) is noteworthy, since some current ABCC1 inhibitors do not exhibit strong selectivity for drug transporters. For example, cyclosporine A inhibits a wide variety of ABC transporters^8,9,13^ and, as demonstrated in this and another study^17^, MK571 also inhibits ABCB1-mediated drug efflux. In contrast, our current study suggests that β-cholanic acid may have greater specificity, with no effect on doxorubicin accumulation in ABCB1-expressing cells. Additional studies are required to assess the efficacy of bile acids to augment efflux of a variety of chemotherapy drugs in chemoresistant cells, including cell lines genetically engineered to overexpress specific ABC transporters. In particular, expression vectors should include ABC transporters previously implicated in chemotherapy resistance and drug disposition, including additional ABCC isoforms^46^ and ABCG2^47^.

Our study also showed that bile acids have no effect on doxorubicin accumulation into drug-sensitive tumour cells that do not express ABCC1. This is an important observation, since bile acids are known to affect lipid solubility^19^ and this could increase plasma membrane permeability towards doxorubicin and other chemotherapy agents in tumour cells.

While in some cases, cells treated with very low doses of doxorubicin did exhibit higher survival fractions than their untreated counterparts, this is likely an artifact of the clonogenic assay. While care was taken to ensure equal plating of cells between samples, in some cases cells spread to the periphery of the plate, where colonies cannot be distinguished clearly. In addition, plating efficiency can vary amongst wells in an experiment. For this reason, only representative clonogenic experiments were presented, while IC50 values were calculated as an average of 3 or more independent clonogenic studies.

Hembruff *et al*.^34^ demonstrated that a majority of MCF-7 cells selected for resistance to anthracyclines and taxanes expressed ABCB1, including cells selected for resistance to docetaxel, paclitaxel or epirubicin. Docetaxel-selected cell lines also expressed ABCC2 at low selection doses [Cells exposed up to an intermediate selection dose were used in our study (MCF-7_TXT10_ cells)]. In contrast, the doxorubicin-resistant cells used in our study predominantly expressed the ABCC1 transporter, with no elevated expression of ABCB1^34^. Since only our MCF-7_DOX2-12_ cell line showed reductions in doxorubicin accumulation in the presence of bile acids, we therefore chose to focus on ABCC1 rather than ABCB1, which is not expressed in MCF-7_DOX2-12_ cells.

We proceeded to further test our hypothesis using two other ABCC1 expressing cell lines, the doxorubicin-resistant H69_AR_ lung cancer cell line, and an ABCC1-transfected cell line (HEK293_MRP1_). For both cell lines, high concentrations of bile acids reduced the IC50 values for doxorubicin by 3–4 fold compared to their respective parental controls. Similar to what was observed in parental MCF-7 cells, bile acids had no effect on doxorubicin sensitivity in parental H69 and HEK293 cells.

Previous studies by other research groups have shown that various bile acids can inhibit ABCC1 transport activity in erythrocytes in the low micromolar range^28,48^. In these studies, the researchers monitored the efflux of a pre-loaded fluorescent molecule from erythrocytes. These studies do provide insight into the effects of bile acids on ABCC1 transporter activity, since erythrocytes are known to express appreciable levels of ABCC1. However, it is not the only ABC transporter present in erythroctyes^29,48–50^. In addition to ABCC1, erythrocytes also express ABCC4 and ABCG2^49,50^. Moreover, our studies are the first to describe the ability of bile acids to affect ABCC1 activity and doxorubicin sensitivity in drug-resistant tumour cells in culture and in cells genetically engineered to overexpress the ABCC1 transporter.

As we have demonstrated here, the potency of the bile acids appears to be lower in terms of their ability to affect doxorubicin retention by inhibiting ABCC1 activity. Where prior studies in erythrocytes have suggested that deoxycholic acid has an IC50 of 16 μM for inhibition of fluorescent substrate efflux, we have observed IC50’s ~10-fold higher for inhibition of ABCC1-mediated doxorubicin efflux from tumour cells. Unlike the previous studies in erythrocytes, our study focussed on nucleated cancer cells which, as seen in many tumour cell lines, are poyploid. Chromosomal duplications could contribute to substantially higher levels of ABC transporter expression in drug-resistant cells, well above those observed in normal erythrocytes. Indeed, a chromosomal region harbouring the *ABCC1* gene (16p13.1) is amplified almost 100-fold in a multidrug-resistant lung cancer cell line^16^. Also, we focussed our study on the effect of bile acids on the ABCC1-mediated transport of doxorubicin, a clinically relevant chemotherapy agent^51–53^. Moreover, we chose to study highly chemoresistant tumour cells, rather than erythrocytes. Nevertheless, the studies in erythrocytes underscore additional effects that bile acids can be expected to exert on normal cell populations within the host. Moreover, our study does not represent the first study on the effects of bile acids on tumour cells. Several groups have attempted to use bile acids and their derivatives as carrier agents for chemotherapy delivery, or even to induce apoptosis^54–56^. However, our observations clearly show that the bile acids in this case are not simply acting as carriers for doxorubicin, since no increase in doxorubicin accumulation was observed in tumour cells not expressing ABCC1 transporters.

In a related study on bile acid efficacy and toxicity, Mrowczynska *et al*. showed that erythrocytes could tolerate very high levels of bile acids, much higher than those used in our study. Additionally, the concentration of β-cholanic acid used in our study has been well documented to inhibit, specifically, the enzymatic activities of Akr1C2 and Akr1C3^31^. Nevertheless, much work remains to be conducted to better understand the systemic effects and clinical toxicities associated with bile acid treatment. Our study also indicates that the concentration of bile acids required to inhibit ABCC1 transport is highly dependent on the model used for study and possibly the level of expression of ABCC1. Further experimentation in animal models would be required to examine the therapeutic efficacy and systemic toxicities of doxorubicin in the presence of various bile acids.

### Future Perspectives

We have demonstrated the ability of bile acids to increase doxorubicin cytotoxicity through the inhibition of ABCC1-mediated doxorubicin transport in various tumour cell lines *in vitro*. However, we do recognize that the affinity of bile acids for ABCC1 is lower than other small molecule ABCC1 inhibitors. Thus, future efforts could be centred on using the structure of β-cholanic acid to design and develop small molecules that have greater affinity for ABCC1, while possibly retaining its minimal *in vivo* toxicity and its ability to inhibit the aldo-keto reductases. However, it is likely that one or more of the chemical properties of β-cholanic acid may change in the hunt to find derivatives with greater ability to inhibit ABCC1 transport activity. Numerous *in vivo* studies, likely in mice or rats, would be necessary to ensure that the desired activities are retained, hopefully without consequent increases in host-related toxicities.

## Materials and Methods

### Cell culture

The MCF-7 breast cancer cell line was originally purchased from the American Type Culture Collection and maintained in high glucose DMEM medium supplemented with 10% FBS and 100 μg/ml streptomycin and 100 units/ml penicillin (Hyclone, Mississauga, Ontario) at 37 °C in 5% CO_2_. For subculturing, cells in T75 Sarstedt flasks were washed once with sterile PBS followed by the addition of 3 ml of a sterile 0.25% Trypsin, 10 mM EDTA solution (Invitrogen). MCF-7 cells were selected for resistance to increasing doses of either doxorubicin (MCF-7_DOX2_ cells) or docetaxel (MCF-7_TXT_ cells) and characterized for expression of ABC transporters as well as other chemotherapy-resistance proteins by Hembruff *et al*. and Veitch *et al*.^30,32^. Doxorubicin- or docetaxel-resistant cells were maintained in media supplemented with the corresponding selection dose of doxorubicin or docetaxel, respectively. Resistant cells were removed from drug-containing media 3–4 days prior to being used in experiments. H69 small cell lung cancer cells, doxorubicin-resistant H69_AR_ small cell lung cancer cells, HEK293 human endothelial kidney cells, and *ABCC1*-transfected HEK293 cells (HEK293_MRP1_) were kindly provided from Dr. Susan Cole of Queen’s University, Kingston, ON, Canada. HEK293 and HEK293_MRP1_ cells were maintained under the same conditions as the MCF-7 cells with the exception that media was supplemented with 500 μg/ml of G418 to maintain selection for clones overexpressing *ABCC1*. H69 cells were maintained under similar conditions, except in RPMI growth media. Additionally, H69 and H69_AR_ cells grow as suspension cultures and consequently did not require the use of trypsin to subculture.

### Doxorubicin accumulation assay

Steady-state doxorubicin retention in cells was measured via flow cytometry, since the drug is fluorescent. Cells were seeded onto 6 well plates at a density of 200 000 cells per well and allowed to adhere. Cells were treated with 2 uM doxorubicin, various concentrations of β-cholanic acid, deoxycolic acid, MK571, probenecid or a combination of these with doxorubicin for a period of 6 h. The concentration range for β-cholanic acid was varied in previous experiments to identify the maximally tolerated dose for MCF-7 cells^30,32^. Deoxycolic acid was tested at a concentration identical to β-cholanic acid, with no observable changes in cell health or morphology for either bile acid. Concentrations for MK571 and probenecid were based on the manufacturer’s recommendation and also showed no discernible morphological effects on the cell population. Cells were collected using trypsin as described above and suspended in 1 ml PBS. Suspended cells were run on an FC500 flow cytometer (Beckman-Coulter) using an FL2 (575 nm) filter to detect doxorubicin fluorescence. Data points shown are the mean of 3 independent experiments and 10 000 events were measured in each experiment.

### Glutathione levels

Glutathione levels in MCF-7_DOX2-12_ whole cell extracts were measured using a glutathione quantification kit from ENZO Life Science, Inc (Brockville, ON). Cells were plated in 10 cm plates at a density of 2 × 10^6^ cells per plate. Cells were pre-treated with the glutamate-cysteine ligase inhibitor buthionine sulphoximine (50 μM for 1 h) to prevent subsequent glutathione synthesis. Cell lysates were collected and glutathione assayed as per the manufacturer’s instructions. Data shown are mean of 3 independent experiments ± SEM.

### Clonogenic assays

Cell viability was measured using a clonogenic assay as previously described^30^. Briefly, cells were plated in 6 well plates at 200 000 cells per well. Cells were treated with increasing concentrations of doxorubicin in the presence or absence of test compounds for 24 h (for example, bile acids). Cells were then collected and incubated in drug-free semi-solid methylcellulose medium until large colonies became visible, if possible (5–10 days). In each experiment, 12 random fields in each well were viewed and the number of colonies counted. IC50 values were calculated by determining the survival fraction relative to untreated control cells or, where relevant, bile acid-treated cells (to examine the effect of bile acids on cellular sensitivity to doxorubicin). Each clonogenic assay was repeated three times and IC50 values reported as means (+/−SEM) for three independent experiments.

### Preparation of whole cell extracts and western blotting experiments

Cells were grown on 10 cm cell culture plates (Sarstedt) and grown to 80% confluency. Cells were lysed and protein extracts prepared as previously described^34^. Briefly, RIPA (Radio- immunoprecipitation Assay) buffer was modified to contain 1% SDS (w/v), 1 × protease inhibitor cocktail (Complete^TM^), 10 μM sodium fluoride, and 2 mM sodium orthovanadate. This buffer was then used to lyse cells and solubilize proteins. Lysates were homogenized and sheared using a 21 gauge needle, and then subjected to centrifugation at 14,000 × g for 5 minutes at 4 °C. The supernatant was then collected and aliquoted for western blotting. Protein content in the aliquots was assayed using a Pierce BCA protein assay prior to the addition of Laemmli sample buffer. The proteins in the samples were then denatured by boiling for 5 min, after which they were loaded on SDS PAGE gels.

ABCC1 protein levels were quantified by immunoblotting 30 μg of cell lysate protein onto a 7% polyacrylamide gel. Proteins were resolved by electrophoresis at 90 V, until the molecular weight range of interest (100–250 kDa) was adequately resolved on the gel. The proteins were then transferred to nitrocellulose membranes using a semi-dry transfer apparatus applying 12 V for 1 hour (Biorad). To detect ABCC1, membranes were first blocked using 5% skim milk in TBST (Tris-buffered saline + 0.1% TWEEN) for 1 hour at room temperature. The membranes were then incubated overnight at 4 °C in primary antibody (monoclonal QCRL-1 Santa Cruz Biotechnology, sc-18835) at a dilution of 1:200 in 5% skim milk in TBST. The secondary antibody [Santa Cruz Biotechnology goat anti-mouse HRPase conjugate (sc-2005) at a 1:10,000 dilution in 5% skim milk in TBST] was applied the following day for 1 hour at room temperature. Images were captured on high-sensitivity film using enhanced chemiluminescence reagent (Santa Cruz). Scanned images of the film were analyzed using AlphaEaseFC 4.0 comparing pixel density levels of the bands relative to the background density of the film. Each band was normalized to a γ-tubulin loading control (Sigma, St. Louis, MO), with the same secondary antibody previously described.

### Data analysis

All graphs were prepared and statistical analysis performed using GraphPad Prism V5.0 software. All data points are representative of the mean ± standard error of mean (SEM). Due to the multi-parametric nature of the data analysis, an ANOVA was chosen as the most suitable method of analysis. Analysis of Variance (ANOVA) tests were performed, assuming normal distribution for all datasets, followed by Bonferoni post hoc testing for significance. Comparisons were made between multiple points, i.e. between control cell lines and their respective chemotherapy- resistant cell lines at various selection doses, as well as comparing differences between treated and untreated cell lines or between transfected or non-transfected cell lines. Differences between samples or cell lines were determined to be significant when P < 0.05.
